# A critical review to traumatic brain injury clinical practice guidelines

**DOI:** 10.1097/MD.0000000000014592

**Published:** 2019-03-01

**Authors:** Bao-shan Di, Min Wei, Wen-juan Ma, Qi Zhang, An-qing Lu, Hu Wang, Yang Niu, Nong Cao, Tian-kang Guo

**Affiliations:** a Gansu Province People's Hospital; b The First Hospital of Lanzhou University; c Anesthesia Department, Traditional Chinese Medicine of Gansu Province; d Evidence-Based Medicine Center, Lanzhou University, Lanzhou, China.

**Keywords:** clinical practice guidelines, quality assessment, traumatic brain injury

## Abstract

The aim of this study was to assess the quality of clinical practice guidelines of traumatic brain injury (TBI) and investigate the evidence grading systems.

A systematic search of relevant guideline websites and literature databases (including PubMed, NGC, SIGN, NICE, GIN, and Google) was undertaken from inception to May 2018 to identify and select TBI guidelines. Four independent reviewers assessed the eligible guidelines using the Appraisal of Guidelines for Research and Evaluation (AGREE II) instrument. The degree of agreement was evaluated with intraclass correlation coefficient (ICC).

From 1802 records retrieved, 12 TBI guidelines were included. The mean scores for each AGREE II domain were as follows: scope and purpose (mean ± SD= 74.2 ± 9.09); stakeholder involvement (mean± SD= 54.6 ± 11.6); rigor of development (mean ± SD=70.1 ± 13.6); clarity and presentation (mean ± SD=78.4 ± 11.5); applicability (mean ± SD= 60.5 ± 13.6); and editorial independence (mean ± SD=61.7 ± 14.8). Ten guidelines were rated as “recommended.” The ICC values ranged from 0.73 to 0.95. Seven grading systems were used by TBI guidelines to rate the level of evidence and the strength of recommendation.

Most TBI guidelines got a high-quality rating, whereas a standardized grading system should be adopted to provide clear information about the level of evidence and strength of recommendation in TBI guidelines.

## 1 Introduction

Traumatic brain injury (TBI) is one of the leading causes of death and disability in both developing and developed countries, with the highest incidence among young people <30 years of age.^[1,2]^ Clinical practice guidelines (CPGs) have been developed by various organizations from different countries to improve patient's outcomes of TBI; the brain trauma community's approach to guideline development has evolved as the science and application of evidence-based medicine advanced.

During the past 20 years, >30 TBI guidelines have been developed and updated from different organizations.^[3]^ However, TBI guidelines vary in quality, comprehensiveness, and grading system, leading to difficulties with standardization of care, adaptation, and implementation. Despite this, a major criticism of the TBI guidelines is that they may not be appropriate for use in all locations due to differences in available resources. Although some previous studies have evaluated quality of existing TBI guidelines, they just have focused on the subsets of TBI severity such as mild TBI only^[3–5]^ or reviewed only a limited number of TBI guidelines,^[6]^ and none of them focused on the grading systems of TBI guidelines adopted, which actually is very important to help guideline users, readers, and stakeholders to understand the confidence of estimate of the effects and the strength of recommendations. Moreover, according to Institute of Medicine (IOM) statement of guideline,^[7]^ some old TBI guidelines (published before 2007) evaluated by these studies had been abandoned in clinical practice as the recommendations were outdated.

Hence, we conducted this study to assess and summarize the quality of all currently available international TBI guidelines by conducting a systematic review using the Appraisal of Guidelines for Research and Evaluation (AGREE) II instrument.^[6]^ We also compared the codes of evidence quality and strength of recommendation among different TBI guidelines.

## 2 Materials and methods

### 2.1 Study design and protocol

This study conducted a comprehensive review of clinical guidelines using the AGREE II instrument. This study was performed in accordance with the guidelines from preferred reporting items for systematic reviews and meta-analyses (PRISMA).^[8]^

### 2.2 Identification of guidelines

Systematic searches were performed in PubMed database from inception to May 31, 2018, combining the term “traumatic brain injury OR TBI” and a filter to identify guideline documents (Practice Guideline [pt] OR Guideline [pt] OR guideline∗ [ti] OR statement [ti], recommendations [ti] OR consensus [ti]). We also searched the websites of guideline development organizations, NICE (https://www.nice.org.uk/guidance) and SIGN (http://www.sign.ac.uk/), and guideline databases such as GIN (http://www.g-i-n.net/) and NGC (https://www.guideline.gov/). Besides, we searched Google Search Engine as well as the references of all the obtained guidelines to include more potential guidelines.

Two reviewers (DBS and WKP) independently evaluated search results to determine inclusion or exclusion of references and extracted the general characteristics of each guideline. Disagreements were resolved by consensus or by consulting the third expert adjudicator (GTK).

### 2.3 Selection of guidelines

The inclusion criteria were as follows: complete guideline text is available in English; guideline contains recommendations regarding TBI interventions; and the guideline should be published after 2007. If the guideline had updates, only the most recent version was assessed. For every guideline ultimately included, we thoroughly searched for accompanying technical and supporting documents to better inform our assessments. The following literatures will be excluded: duplicate guidelines, guidelines for patients, editorials, translations of guidelines, secondary or multiple publications, and short summaries. For multiple versions of guidelines, only the newest guidelines were included in the analysis and the older versions were excluded.

### 2.4 Quality appraisal of guidelines

We used the latest version of the AGREE II instrument to evaluate each TBI CPG meeting our inclusion criteria. According to AGREE II handbook, each CPG was scored on 23 items within 6 domains. Domain 1 (scope and purpose) is divided into 3 items: guideline objectives, health questions, and population application. Domain 2 (stakeholder involvement) is based on 3 items: guideline development group, preferences of target population, and target users. Domain 3 (rigor of development) includes 8 items: systematic methods used to search evidence, criteria for selection, strengths and limitations of the evidence, methods for formulating the evidence, health benefits and side effects of recommendations, explicit links between recommendation and supporting evidence, expert reviewers, and updating guideline for future use. Domain 4 (clarity and presentation) includes 3 items: recommendations are specific and unambiguous, different options for management, and key recommendations. Domain 5 (applicability) includes 4 items: facilitators and barriers, advice/tools to implement recommendations into practice, resources for implications, and auditing criteria. Domain 6 (editorial independence) is based on 2 items: editorial independence from the funding body and conflicts of interest of the guideline development members.

In this study, each TBI guideline was scored by 4 independent reviewers (DBS, WKP, MWJ, and M.W.J) according to AGREE II user manual. Among the 4 reviewers, DBS and WKP are senior doctors of neurosurgery; MWJ and LY are methodologists in guideline development. Besides, YL had rich experiences in the application of AGREE II and published a study about using AGREE II to assess clinical guidelines,^[9,10]^ DBS and WKP were trained to use the AGREE II instrument through the online tutorials on the AGREE website.

The user manual defines each item and assists the user in determining a guideline's score for that item. Items were scored based on a scale ranging from 1 (absence of item) to 7 (item is reported with exceptional quality). Domain scores were calculated by summing item scores within each domain from each reviewer, and then standardizing them as a percentage of the maximum possible score. AGREE II protocol states that no overall score is calculated to determine if a CPG is recommended or not recommended. Instead, guidelines in this study were recommend if the guidelines have >4 domains scoring >50%.^[6]^

### 2.5 Strength of recommendation and level of evidence

The strength of recommendations and level of evidence of each TBI guideline were extracted if these guidelines adopted evidence grading systems.

### 2.6 Data analysis

We performed a descriptive statistics analysis using the calculation of the total score by each reviewer and the score per domain. The number of recommendations and the percentage distributions among quality of evidence and strength of recommendation classes were determined. Agreement between each reviewer's scores was tested using a 2-way ANOVA with single-rater 2-way intraclass correlation coefficients (ICCs) with 95% confidence interval (CI) for each domain across all guidelines.^[11]^ According to a previous study,^[12]^ the degree of agreement between 0.01 and 0.20 was deemed minor, 0.21 to 0.40 fair, 0.41 to 0.60 moderate, 0.61 to 0.80 substantial, and 0.81 to 1.00 very good. A value of *P* <.05 denoted statistical significance. All tests were 2-sided. Statistical analyses were conducted using SPSS version 19.0 (SPSS Inc., Chicago, IL).

## 3 Results

### 3.1 Study selection

**Figure 1:**
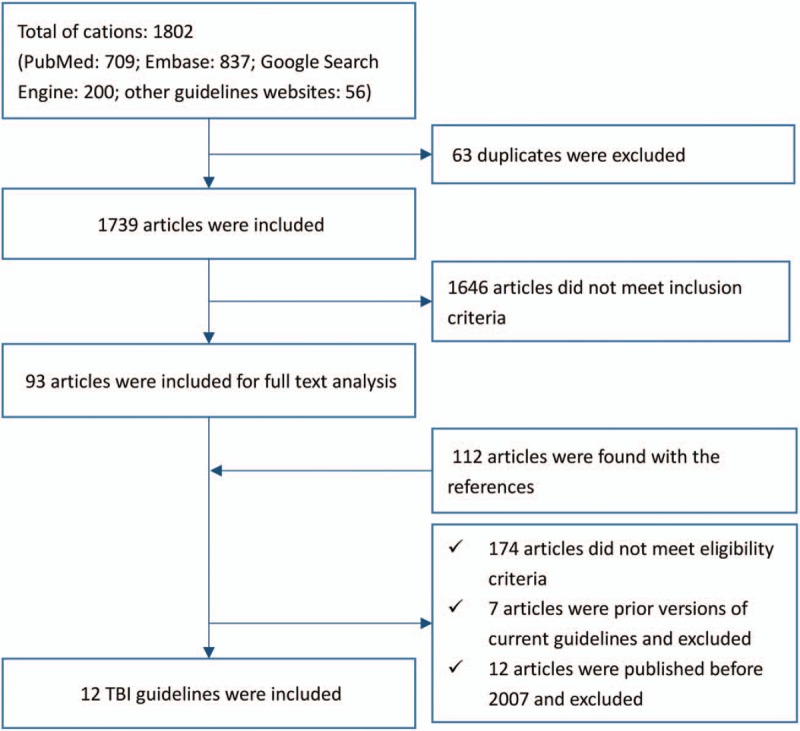
Flow chart of selecting TBI guidelines.

The initial search strategy identified 1802 titles and abstracts, 63 of which were removed for duplicates. From these, 1646 were excluded after reviewing abstracts. A reference and citation analysis was performed on the remaining 93 articles yielding an additional 112 abstracts. Full-text analysis was then performed on a total of 205 articles of which only 12^[13–24]^ met inclusion criteria (Fig. 1).

### 3.2 CPG characteristics

Overall, 12 TBI guidelines were included in this analysis (Table 1), representing 12 different organizations and spanning several countries on 4 continents. Of these 12 TBI guidelines, all of them^[22,28,4,29–43]^ were developed in high-income countries. The CPGs evaluated covered the full scope of adult and pediatric populations with 1 covering pediatric patients,^[20]^ 5 for adults patients,^[14,15,19,21,24]^ and 4 covering both populations.^[13,16,18,22]^ Regarding the severity of TBI, one-fourth of the guidelines were developed for minor or mild TBI,^[15,18,23]^ another third covered severe TBI,^[17,20,24]^ and the rest were developed for all levels of TBI severity.^[13,14,16,19,21,22]^ The majority (7) of CPGs focused on the early management of TBI.^[13,14,16,20–23]^ Of the 24 assessed CPGs, 4 were developed by professional organizations,^[13,15,23,24]^ 2 were developed by nonprofit organizations,^[16,20]^ 2 by international committees,^[18,21]^ and another 4 by national institutes or government organizations.^[14,17,19,22]^ The detailed information of included guidelines was shown in Table 1.

**Table 1:**
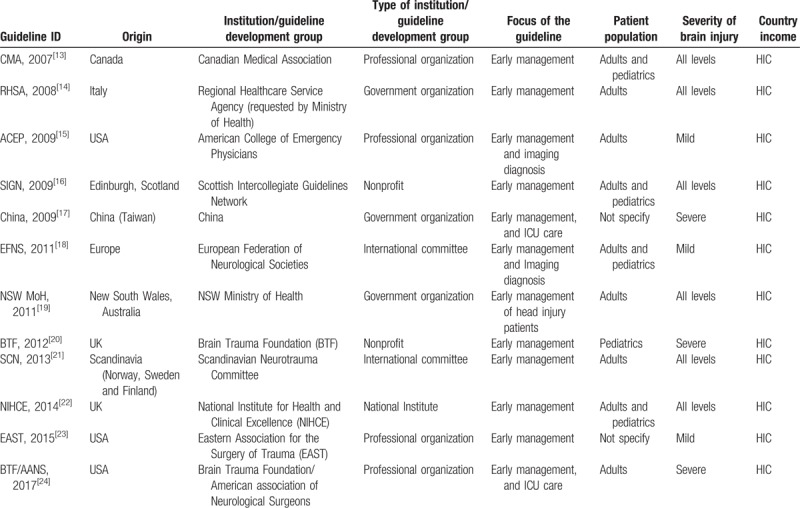
The characteristics of included TBI guidelines.

### 3.3 CPG quality assessment (AGREE)

#### 3.3.1 Consistency

The ICC values, which indicate the overall agreement between reviewers, generally received higher reliability scores. The ICC values for TBI guidelines appraisal using the AGREE II ranged from 0.73 to 0.95 (Table 2). The ICCs for the AGREE appraisal conducted by the 4 reviewers were lowest in the “applicability” domain (0.73) but higher in the other 5 domains (all ≥0.75), which indicated the intrareviewer item score agreement was good. Domain scores of the AGREE II quality assessment are illustrated in Table 2.

**Table 2:**
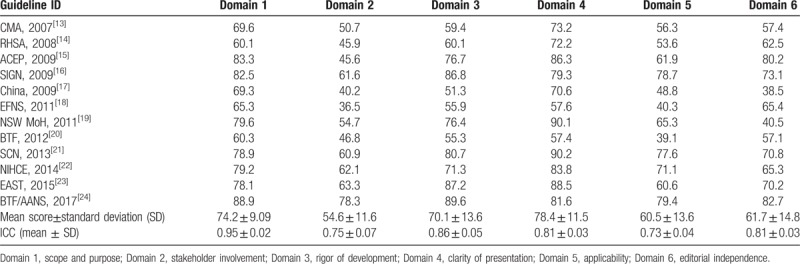
AGREE score by domain of each TBI guidelines.

#### 3.3.2 Domain 1—scope and purpose

This domain focuses on the overall objectives, expected benefits or outcomes and target population of the guidelines. The mean score of TBI guidelines in this domain is 74.2 with a SD of 9.09, and all guidelines scored >50. The lowest score was 60.1 which was from guidelines for the treatment of minor and severe TBI (RASH, 2008). The highest score was 88.9, from Brain Trauma Foundation and American association of Neurological Surgeons (BTF/AANS, 2017).

#### 3.3.3 Domain 2—stakeholder involvement

This domain contains items on the involvement degree of professional members, consideration of the views and preferences of the target population, and the definition of target users. Scores fluctuated remarkably with a mean score±SD of 54.6 ± 11.6. Five (38%) TBI guidelines scored <50%, of which the lowest was 40.2 from China (China, 2009).

#### 3.3.4 Domain 3—rigor of development

This domain investigates the method and process of evidence search, grading, summary, and the formulation of the recommendations. The mean score and SD for this domain was 70.1 ± 13.6. All TBI guidelines scored >50%, of which the lowest was 51.3 from China (China, 2009).

#### 3.3.5 Domain 4—clarity of presentation

This domain addresses the presentation and format of guidelines. The mean score and SD in this domain was 78.4 ± 11.5. The lowest score was 57.4 from Brain Trauma Foundation (BTF, 2012).

#### 3.3.6 Domain 5—application

This domain evaluates the consideration of facilitators or barriers when implementing the guidelines and the monitoring criteria. The mean score and SD of this domain was 60.5 ± 13.6, among which 3 TBI guidelines scored <50, with the lowest score of 39.1 from Brain Trauma Foundation (BTF, 2012).

#### 3.3.7 Domain 6—editorial independence

This domain considers funders and competing interests of experts involved in guideline development. The mean score and SD of this domain was 61.7 ± 14.8 and 3 scored <50%. The score among these guidelines varied a lot, the lowest score of 38.5 points came from China (China, 2009) and the highest score was 82.7, from Brain Trauma Foundation and American association of Neurological Surgeons (BTF/AANS, 2017).

#### 3.3.8 Overall assessment

This assessment concerns “the rating of body quality of the guidelines and whether the guideline would be recommended for use in practice.” According to the appraisal of the individual domains and overall scores, 10 TBI guidelines had >4 domains scored >50, and rated as “recommended” by the appraisers (Table 2).

#### 3.3.9 Level of evidence and strength of recommendation

Of the 12 included TBI guidelines, all of them were deemed evidence-based; 11 guidelines used 7 grading systems to rate the level of evidence and the strength of recommendation, among which 4^[21–24]^ adopted GRADE system (EAST, 2015; NIHCE, 2014; SCN, 2013; BTF/AANS, 2017), 2^[17,18]^ used SIGN system^[11]^ (China,2009; SIGN,2009), 1 used NHMRC system (NSW MoH, 2011),^[19]^ 1 used^[20]^ USPSTF system (BTF, 2012), 1^[14]^ used PNLG system (RHSA, 2008), 1^[18]^ used AAN system (EFNS,2011) and 1^[15]^ used ACEP system (ACEP, 2009). However, the codes of level of evidence and strength of recommendation in different grading systems varied a lot (Table 3  ).

**Table 3:**
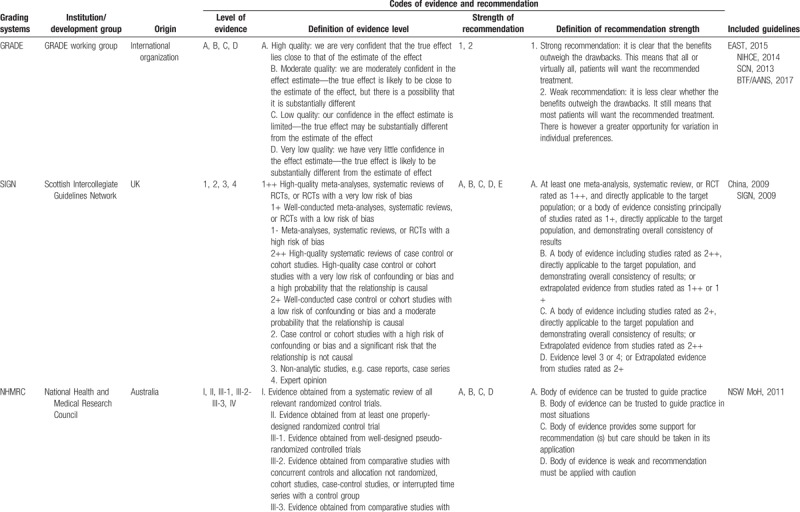
Grading systems used in the included guidelines.

**Table 3 (Continued):**
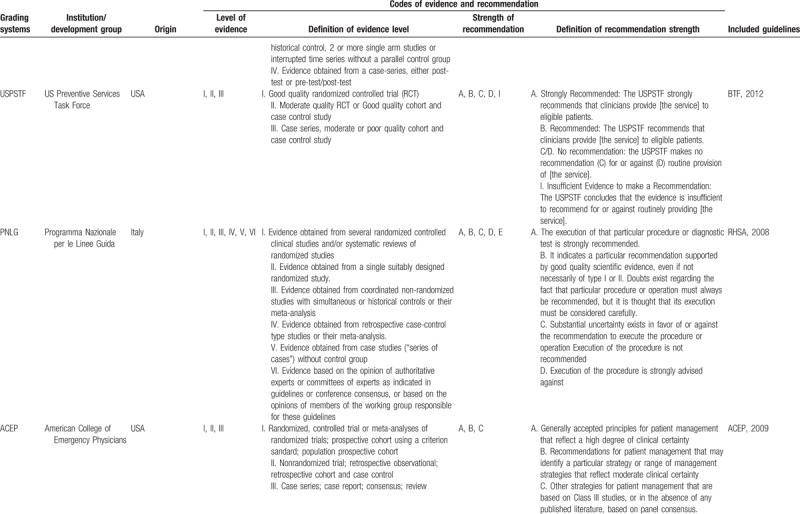
Grading systems used in the included guidelines.

**Table 3 (Continued):**
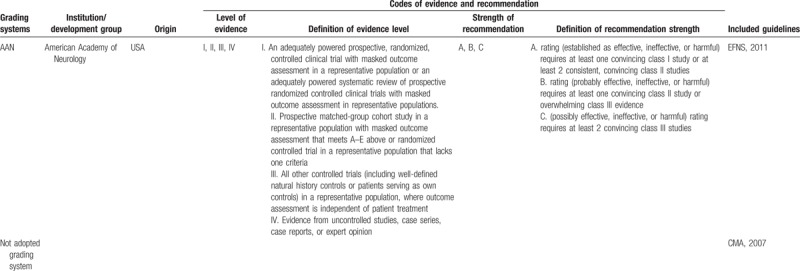
Grading systems used in the included guidelines.

## 4 Discussion

This critical review investigated the quality of TBI guidelines published after 2006. Although there exist some TBI guidelines published before 2006,^[25–28]^ and still were not updated, the recommendations in the guidelines had been outdated and could not be used in practice according to IOM statements of clinical guidelines.^[7]^ Hence, we did not include these guidelines in this review.

Our study included 12 TBI guidelines; across all TBI guidelines, the highest mean scores were achieved in clarity and presentation, scope and purpose, and rigor of development, whereas the main weaknesses across TBI guidelines were stakeholder involvement, applicability, and editorial independence. Brain Trauma Foundation/American association of Neurological Surgeons (BTF/AANS, 2017), Scandinavian Neurotrauma Committee (SCN, 2013), and Eastern Association for the Surgery of Trauma (EAST, 2015) were the 3 CPGs with best results. All of the TBI guidelines evaluated in this study were developed by high-income countries (HICs) and are therefore minimally applicable in resource-limited settings. Besides, the distribution of level of evidence and strength of recommendations varied significantly among different TBI guidelines.

Overall, the strong scores in the clarity and presentation, scope and purpose, and rigor of development domains have been reported in other systematic reviews evaluating TBI CPGs.^[4,29–30]^ This is likely attributed to the scientific rigor of developing a guideline, which typically involves a highly methodical approach.^[31]^ In general, the guidelines that were more recently developed or updated, and those that had undergone numerous updates, most consistently demonstrated the highest quality by AGREE II scores.

Our analysis indicates an overall improvement in the above domains in the most current CPGs, consistent with other studies.^[21–24]^ In a 2011 critical review of mild TBI guidelines by Tavender et al,^[4]^ the NSW 2006 TBI guideline got a worse score of AGREE II in all domains with the exception of scope and purpose domain when compared with the updated 2011 version.^[19]^ It is noteworthy to mention that new version of TBI guidelines also has the advantage of newer and more rigorous evidence-based medicine in addition to the availability of designing guidelines around the AGREE II format. Nevertheless, a frequently criticized area in our results, within the rigor of development domain, was the lack of procedures for updating the guidelines for quality improvement. Given the trend toward improved guideline quality with newer revisions, development of a quality improvement list may help to ensure quality of future TBI guidelines.

The AGREE II result may also associate with reporting of key information in guidelines, which indicated more attention should be paid to improve the reporting quality of guidelines. In 2016, the AGREE working group developed a new checklist for improving the reporting quality of CPGs,^[32]^ which might be referred by TBI guidelines developers in the future.

Older reviews have demonstrated limited stakeholder involvement in TBI guideline development, a trend that persisted in new versions.^[4,33]^ Although there have been progressive improvement in guideline development, the domains of stakeholder involvement, applicability, and editorial independence remain weak, specifically when it comes to piloting interventions, addressing potential costs, barriers to implementation, and auditing for quality improvement. Recent studies suggested that successful implementation of guidelines could improve patient outcomes^[34–36]^; however, applicability of guidelines to a given locale based on factors, such as availability and cost of resources, provider skills, and population needs and values, is critically important for successful implementation of guidelines in a manner that will improve patient care. Consideration of stakeholder involvement and applicability are imperative considering these domains are intrinsically associated with CPG implementation and translation to other settings such as LMICs.

It has been suggested that adaptation of existing guidelines to local situations may be a more valid and cost-effective means of achieving high-quality guidelines worldwide.^[37]^ However, the various codes of evidence level and strength of recommendation could bring challenges to reach the target. As we know, most guidelines, especially evidence-based guidelines, applied grading systems to communicate clear message, quickly and concisely so as to help guideline users, readers, and stakeholders to understand the confidence of estimate of the effects and the strength of recommendations easily. The confidence of estimate of the effects reflects the extent to which confidence in an estimate of the effect is adequate to support a particular recommendation. And the strength of guideline recommendation reflects the extent of collective confidence that adherence to the recommendation will do more good than harm.^[38,39]^ However, in our study, different grading systems with various systems of codes were used to communicate grades of evidence and recommendations in TBI guidelines, which could confuse the guideline users when using these guidelines. Therefore, a standardized grading system should be established to provide a clear information about the level of evidence and the strength of recommendation for TBI guidelines users, and the good news is that we find some guideline organizations such as Scottish Intercollegiate Guidelines Network (SIGN) and National Health and Medical Research Council (NHMRC) begin to adopt GRADE system instead of old systems in their new version of guideline development handbooks.^[40,41]^

### 4.1 Strengths and limitations

Our overall findings have some strengths. First, our authors were from different background consisting of clinical experts and methodologists with extensive experience in evaluating clinical guidelines, which improved the reliability of our findings. Second, different domains have been appropriately weighed to derive overall assessment and recommendation. Nonetheless, our study also has limitations. Exclusion of guidelines published in languages other than English, or other forms (i.e., books, booklets, or government documents), might have resulted in under-representation of guidelines from less developed countries. Third, AGREE II instrument focused on methods of guideline development and the transparency of reporting, but could not assess potential impacts of recommendations on patient's outcomes.^[42,43]^ Furthermore, our study could not establish the causality between the poor performance and the characteristics of TBI guidelines, matching current guidelines to future guideline updates (in a cohort study) would allow for better assessment of guideline quality than did our cross-sectional assessment.

## 5 Conclusions

Most TBI guidelines got a high-quality rating. The high-quality domains were achieved in clarity and presentation, scope and purpose, and rigor of development. Our findings called for a standardized grading system to provide a clear information about the level of evidence and the strength of recommendation in TBI guidelines.

## Acknowledgments

We thank Jinhui Tian and Kehu Yang (Evidence-Based Medicine Center of Lanzhou University) for supporting the methodology and Liang Yao (Hong Kong Baptist University) for providing assistance with editing the final article.

## Author contributions

LAQ and WKP had full access to all of the data in the study and taken responsibility for the integrity of the data and the accuracy of the data analysis. CN and GTK contributed to drafting and critical revision of the article; DBS, WKP, and MWJ contributed to the data collection, AGREE process, and data analysis; DBS and GTK contributed to the study design and critical review. All authors contributed to the interpretation of study data and critically reviewed and approved the article before submission.

**Conceptualization:** Anqi Lu.

**Data curation:** Baoshan Di, Kongping Wei, Wenjuan Ma.

**Methodology:** Tiankang Guo.

**Project administration:** Yang Niu.

**Resources:** Qi Zhang.

**Software:** Kongping Wei, Wenjuan Ma.

**Supervision:** Anqi Lu, Hu Wang, Nong Cao, Tiankang Guo.

**Writing – original draft:** Baoshan Di.

**Writing – review and editing:** Baoshan Di, Kongping Wei, Wenjuan Ma, Tiankang Guo.

## References

[1] Hyder A Wunderlich C Puvanachandra P The impact of traumatic brain injuries: a global perspective. NeuroRehabilitation 2007;22:341–53. 18162698

[2] Bruns J Hauser W The epidemiology of traumatic brain injury: a review. Epilepsia 2003;44:2–10. 10.1046/j.1528-1157.44.s10.3.x 14511388

[3] Patel A Vieira MMC Abraham J Quality of the development of traumatic brain injury clinical practice guidelines: a systematic review. PLoS One 2016;11:e0161554. 27583787 10.1371/journal.pone.0161554 PMC5008729

[4] Tavender E Bosch M Green S Quality and consistency of guidelines for the management of mild traumatic brain injury in the emergency department. Acad Emerg Med 2011;18:880–9. 21843224 10.1111/j.1553-2712.2011.01134.x

[5] Rusnak M Mauritz W Lecky F Evaluation of traumatic brain injury guidelines using AGREE instrument. Bratisl Lek Listy 2008;109:374–80. 18837249

[6] Brouwers M Kho M Browman G AGREE II: advancing guideline development, reporting and evaluation in health care. CMAJ 2010;182:E839–42. 20603348 10.1503/cmaj.090449 PMC3001530

[7] Steinberg E Greenfield S Wolman DM Clinical Practice Guidelines We Can Trust. Washington, DC: National Academies Press; 2011. 24983061

[8] Moher D Liberati A Tetzlaff J Preferred Reporting Items for Systematic Reviews and Meta-Analyses: The PRISMA Statement. PLoS Med 2009;6:e1000097. 19621072 10.1371/journal.pmed.1000097 PMC2707599

[9] Chen YL Yao L Xiao XJ Quality assessment of clinical guidelines in China: 1993–2010. Chin Med J 2012;125:3660–4. 23075720

[10] Yao L Chen Y Wang X Appraising the quality of clinical practice guidelines in traditional Chinese medicine using AGREE II instrument: a systematic review. Int J Clin Pract 2017;71:e12931. 10.1111/ijcp.12931 28382763

[11] Shrout P Fleiss J Intraclass correlations: uses in assessing rater reliability. Psychol Bull 1979;86:420–8. 18839484 10.1037//0033-2909.86.2.420

[12] Alonso-Coello P Irfan A Sola I The quality of clinical practice guidelines over the last two decades: a systematic review of guideline appraisal studies. Qual Saf Health Care 2010;19:e58. 21127089 10.1136/qshc.2010.042077

[13] Hebb M Clarke D Tallon J Development of a provincial guideline for the acute assessment and management of adult and pediatric patients with head injuries. Can J Surg 2007;50:187–94. 17568490 PMC2384275

[14] Rusticali B Villani R Treatment of minor and severe traumatic brain injury. National reference guidelines. Minerva Anestesiol 2008;74:583–616. 18854800

[15] Jagoda A Bazarian J Bruns J Clinical policy: neuroimaging and decisionmaking in adult mild traumatic brain injury in the acute setting. J Emerg Nurs 2009;35:e5–40. 19285163 10.1016/j.jen.2008.12.010

[16] Scottish Intercollegiate Guidelines Network. Early Management of Patients With a Head Injury: A National Clinical Guideline. 1st ed. Edinburgh: Scottish Intercollegiate Guidelines Network; 2009 Available at: http://www. sign.ac.uk/pdf/sign110.pdf Accessed April 4, 2018.

[17] Liao K Chang C Chang H Clinical practice guidelines in severe traumatic brain injury in Taiwan. Surg Neurol 2009;72:S66–73. 19818476 10.1016/j.surneu.2009.07.004

[18] V Vos PE Alekseenko Y Battistin L Mild traumatic brain injury. Eur J Neurol 2012;19:191–8. 22260187 10.1111/j.1468-1331.2011.03581.x

[19] NSW Ministry of Health. Adult Trauma Clinical Practice Guidelines: Initial Management of Closed Head Injury in Adults. 2nd ed. 2011;Sydney: NSW Institute of Trauma and Injury Management, 1–129.

[20] Kochanek P Carney N Adelson P Guidelines for the acute medical management of severe traumatic brain injury in infants, children, and adolescents—second edition. Pediatr Crit Care Med 2012;13 Suppl. 1:S1–2. 22217782 10.1097/PCC.0b013e31823f435c

[21] Undén J Ingebrigtsen T Romner B Scandinavian guidelines for initial management of minimal, mild and moderate head injuries in adults: an evidence and consensus-based update. BMC Med 2013;11:50. 23432764 10.1186/1741-7015-11-50 PMC3621842

[22] National Institute for Health and Clinical Excellence: Guidance. Head Injury Triage, Assessment, Investigation and Early Management of Head Injury in Inants, Children and Adults. 1st ed. London: National Institute for Health and Clinical Excellence: Guidance; 2014 Available at: http://www.ncbi.nlm.nih.gov/books/NBK53036/pdf/Bookshelf_NBK53036.pdf Accessed April 4, 2018. 25340248

[23] Seamon MJ Haut ER Van Arendonk K An evidence-based approach to patient selection for emergency department thoracotomy: a practice management guideline from the Eastern Association for the Surgery of Trauma. J Trauma Acute Care Surg 2015;79:159–73. 26091330 10.1097/TA.0000000000000648

[24] Carney N Totten AM O’Reilly C Guidelines for the management of severe traumatic brain injury, fourth edition. Neurosurgery 2017;80:6–15. 27654000 10.1227/NEU.0000000000001432

[25] Schutzman S Barnes P Duhaime A Evaluation and management of children younger than two years old with apparently minor head trauma: proposed guidelines. Pediatrics 2001;107:983–93. 11331675 10.1542/peds.107.5.983

[26] Kamerling S Lutz N Posner J Mild traumatic brain injury in children: practice guidelines for emergency department and hospitalized patients. Pediatr Emerg Care 2003;19:431–40. 14676497 10.1097/01.pec.0000092590.40174.1f

[27] Piek J Guidelines for the pre-hospital care of patients with severe head injuries. Intensive Care Med 1998;24:1221–5. 9876987 10.1007/s001340050748

[28] The management of minor closed head injury in children. Committee on Quality Improvement, American Academy of Pediatrics. Commission on Clinical Policies and Research, American Academy of Family Physicians. Pediatrics 1999;104:1407–15. 10585999

[29] Berrigan L Marshall S McCullagh S Quality of clinical practice guidelines for persons who have sustained mild traumatic brain injury. Brain Injury 2011;25:742–51. 21604931 10.3109/02699052.2011.580317

[30] Grimmer K Dizon J Milanese S Efficient clinical evaluation of guideline quality: development and testing of a new tool. BMC Med Res Methodol 2014;14:63. 24885893 10.1186/1471-2288-14-63 PMC4033487

[31] Qaseem A The development of clinical practice guidelines and guidance statements of the American College of Physicians: summary of methods. Ann Intern Med 2010;153:194. 20679562 10.7326/0003-4819-153-3-201008030-00010

[32] Irving G The AGREE Reporting Checklist is useful for assessing the quality of clinical practice guideline development. BMJ 2016;353:i202711. 10.1136/bmj.i2027 27071697

[33] Alarcon J Rubiano A Chirinos M Clinical practice guidelines for the care of patients with severe traumatic brain injury. J Trauma Acute Care Surg 2013;75:311–9. 23887565 10.1097/TA.0b013e3182924bf8

[34] Lee J Rittenhouse K Bupp K An analysis of Brain Trauma Foundation traumatic brain injury guideline compliance and patient outcome. Injury 2015;46:854–8. 25661105 10.1016/j.injury.2014.12.023

[35] Fakhry S Trask A Waller M Management of brain-injured patients by an evidence-based medicine protocol improves outcomes and decreases hospital charges. J Trauma 2004;56:492–500. 15128118 10.1097/01.ta.0000115650.07193.66

[36] Griesdale D Örtenwall V Norena M Adherence to guidelines for management of cerebral perfusion pressure and outcome in patients who have severe traumatic brain injury. J Crit Care 2015;30:111–5. 25179411 10.1016/j.jcrc.2014.07.026

[37] Fervers B Burgers J Haugh M Adaptation of clinical guidelines: literature review and proposition for a framework and procedure. Int J Qual Health Care 2006;18:167–76. 16766601 10.1093/intqhc/mzi108

[38] Atkins D Best D Briss PA Grading quality of evidence and strength of recommendations. BMJ 2004;328:1490. 15205295 10.1136/bmj.328.7454.1490 PMC428525

[39] Guyatt GH Oxman AD Vist GE GRADE: an emerging consensus on rating quality of evidence and strength of recommendations. BMJ 2008;336:924–6. 18436948 10.1136/bmj.39489.470347.AD PMC2335261

[40] Harbour RT Forsyth L Scottish Intercollegiate Guidelines Network. SIGN 50: A Guideline Developer's Handbook. Scottish Intercollegiate Guidelines Network 2014;Available at: http://www.sign.ac.uk/guidelines/fulltext/50/. Accessed April 3, 2018.

[41] NHMRC Guideline Handbook. Available at: https://www.nhmrc.gov.au/guidelines-publications/information-guideline-developers/resources-guideline-developers Accessed April 3, 2018.

[42] Vlayen J Aertgeerts B Hannes K A systematic review of appraisal tools for clinical practice guidelines: multiple similarities and one common deficit. Int J Qual Health Care 2005;17:235–42. 15743883 10.1093/intqhc/mzi027

[43] Watine J Friedberg B Nagy E Conflict between guideline methodologic quality and recommendation validity: a potential problem for practitioners. Clin Chem 2006;52:65–72. 16391328 10.1373/clinchem.2005.056952

